# How Ice Rink Locations Affect Performance Time in Short-Track Speed Skating

**DOI:** 10.3389/fpsyg.2022.854909

**Published:** 2022-05-27

**Authors:** Lixin Sun, Tianxiao Guo, Fei Liu, Kuan Tao

**Affiliations:** ^1^School of Sports Engineering, Beijing Sport University, Beijing, China; ^2^School of Sports Science, Beijing Sport University, Beijing, China

**Keywords:** speed skating, short track, geographical race factors, race analysis, performance

## Abstract

**Purpose:**

To accurately provide evaluations on how match performance for elite skaters in short track speed skating developed, and whether geographical factors of ice rink locations should be considered apart from technical abilities. We created a dataset containing competition records from the 2013–14 to 2020–21 seasons (500 m event) on the official website.

**Methods:**

One-way ANOVA was applied to statistically analyze whether the best performance times exhibited significant differences in varied hosting cities. Performance–time matrix and multivariate regression model were further established to quantitatively explain how geographical factors (longitude, latitude, altitude, and barometric pressure) affected performance.

**Results:**

Our findings firstly confirmed that the fastest 500 m finishing times varied due to the hosting cities (*P* = 0.008) and showed that venue locations could boost or impair performance time with the maximum range of 3.6 s. Meanwhile, latitude (slightly over 46° when performance is maximized) was the most influential factor to account for the performance–time difference in different ice rink locations according to the multivariate regression model, though altitude (1,225 meters when performance is maximized) was also important.

**Conclusions:**

In this perspective, elite skaters should check the geographical factors of the venues before they participated in the upcoming competitions, assess the real strength of their rivals, and adopt flexible tactics during training sessions.

## Highlights

– Venue locations have a significant impact on performance time for short track speed skaters, which could boost or impair with a maximum effect of 3.6 s.
– Elite skaters should check information about latitude and barometric pressure in the venue and adopt flexible tactics during training sessions while preparing for the upcoming competition.

## Introduction

Performance time for elite skaters in short-track speed skating involves technical abilities, such as strength, quality, motor behavior (Konings et al., 2016; Menting et al., 2019), and race tactics (Bullock et al., 2008). These abilities constitute critical components to elevate performance and lower injury risks during training and competition and attract researchers to study the functionalities of specific muscles. For instance, training should be arranged to minimize the risk of causing fatigue asymmetry of the gluteus maximus muscle in skaters (Konieczny et al., 2020), and the decreased muscle activity of the tibialis anterior and rectus femoris shows the highest relationship with the reduction in skating speed both straight or in the curve (Felser et al., 2016). Interestingly, geographical factors of the venues also significantly affect performance time. In Olympic Games or World Cup Series, the record for men's 500 m short track speed skating event in Salt Lake City was 39.505 s, which was much faster than that in Marquette (40.651 s), as the difference between the altitude above the sea level varies more than 1,000 m, from 1,288 m in Salt Lake City and 203 m in Marquette. Similar observations were obtained for women's 500 m events, the difference for the best performance time reaches approximately 0.67 s from 41.936 s in Salt Lake City to 42.597 s in Dresden (altitude difference is more than 100 m as well). The acute effects of altitude and hypoxia have multidimensional influences on exercise performance (Chapman et al., 2010), especially for racing events in short-track speed skating because the partial pressure of oxygen is proportionally reduced by air density. Meanwhile, the ascent to altitude results in a fall in barometric pressure (Malashenkova and Romova, 2017), which impairs the maximal performance because a decline in atmospheric oxygen activates a maladaptive signaling cascade that reduces oxygen availability to the brain. The type of rink (open vs. closed), on the other hand, is also reported to affect variability among athletes between competitions (Noordhof et al., 2016).

Although current studies focus on energy expenditure that is related to pacing strategy in winter sports, performance time analysis with respect to varied venues is still insufficient. Furthermore, energy consumption is due to physiological processes, including nutritional requirements in high-intensity training periods (Meyer et al., 2011) and the effect of high altitude on performance with a moderate hydration control intervention for alpine skiing athletes (Hydren et al., 2013). From the perspective of performance, a study showed that the reduction of air pressure at higher altitudes increased the medal-winning chances by 10% in long-track speed skating events (Noordhof et al., 2016), and the variability in performance times was similar to that of athletes when air resistance limits speed. Also, the impact of different competitive environments, which consisted of the stage of the competition, time qualification, number of competitors per race, etc., alter the pacing decisions of elite short track skaters (Konings and Hettinga, 2018a,b), in particular in the initial phase of the match.

Despite few works of literature on the considerations of geographical factors for ice rinks, several questions still arise that are worthy to be addressed. (1) Are there any significant differences in performance time when skaters compete in venues with different geographical properties? (2) What is the reference time (indicating the time at the basal value which was caused by geographical factors) for ice rink locations in a variety of cities? (3) What is the quantitative relation between the differences in reference time and multiple geographical factors?

Therefore, the purpose of our study was to quantitatively investigate the effects that different competition venue locations exerted on skating speed for elite short-track speed skaters. Firstly, we tested via the international competitions from the 2013–14 to 2020–21 seasons whether venues with different geographical properties had a significant impact on performance time. Secondly, reference times between competitions held from the intersection of any two cities were computed and pair-wisely compared. Finally, the altitude of venues and the barometric pressure along with the longitude and latitude of the hosting city were obtained and used as input variables to estimate the relation between performance time and geographical factors.

## Methods

### Data Acquisition

In order to investigate whether ice rink locations significantly influenced performance time, 24,330 records including men's and women's competition events from World Cup Series/Championships/Olympic Games from the 2013–14 to 2020–21 season (500 m event) were abstracted from International Skating Union (ISU) website. Data abstraction included distance classifications, competition names, groups, player information, and performance times, as well as arena demographics such as including longitude, latitude, altitude, and barometric pressure.

Cities that hosted at least four competitions were selected from the dataset, and 22 cities were thus obtained, including Beijing, Bormio, Calgary, Dordrecht, Salt Lake City, etc., which have distinct altitudes, barometric pressures, longitudes, and latitudes. Although the self-built dataset contained records from the 2013–14 to 2020–21 seasons, data later than the 2016–17 season were extracted for further analysis in terms of 500 m short-track speed skating, considering that both training techniques and tactics were constantly developing for athletes, and the time span starting from the year around PyeongChang Winter Olympic was more worthwhile to be analyzed. Meanwhile, the fastest finishing time was the major index to reflect on the influence of geographical factors, as it represents the maximal ability.

### Statistical Analysis

We collected the fastest finishing times in 500-m events among each city with the criteria that at least four matches were held. One-way ANOVA was conducted via SPSS (version 16.0) to confirm whether ice rink locations affect performance times by testing the statistical difference between the fastest times. The one-way ANOVA was applied to the dataset, and the fastest finishing time in all the rounds of 500-m events in different hosting cities was selected, including Seoul, Dordrecht, Salt Lake City, Dresden, Shanghai, and Montreal. The results illustrated that the *F* and *F* critical values were equal to 3.797 and 2.503, respectively (with *P* = 0.008 < 0.05), which implied that there were significant statistical differences between best performance times for skaters who competed in different cities and thus laid the foundation for further race factors analysis.

### Derivation of the Performance-Time Difference Matrix

Calculation procedures consisted of four steps: (1) listing all cities that held short track speed skating competitions since 2016 and screening out athletes who participated in two competitions held in different cities, thus with 717 players and 22 cities obtained. (2) Extracting the best records of those skaters and calculating the mean performance times for each city. (3) Building a 22 × 22 performance-time difference matrix to compare the reference time between venue locations with varied geographical race factors. (4) Visualizing the matrix via Python 3.7 so that the diversity of performance-time differences could be impressively observed.

### Multivariate Regression Model

A multivariate regression model was further established to explore the quantitative relation between performance time and race factors. The geographical information of hosting cities, such as longitude, latitude, altitude, and barometric pressure, was gathered as input variables to represent geographical venue-specific factors (Noordhof et al., 2016) and correlation analysis was performed. Note that only those data with the absolute value of differential barometric pressure larger than 7 kPa were incorporated into the regression model because this race factor was highly correlated with altitude that could intuitively affect the somatic function of athletes, and as a result, skaters competing in ice rinks at high altitude led to greater performance.

## Results

### Visualization of the Performance–Time Difference Matrix

**Figure 1:**
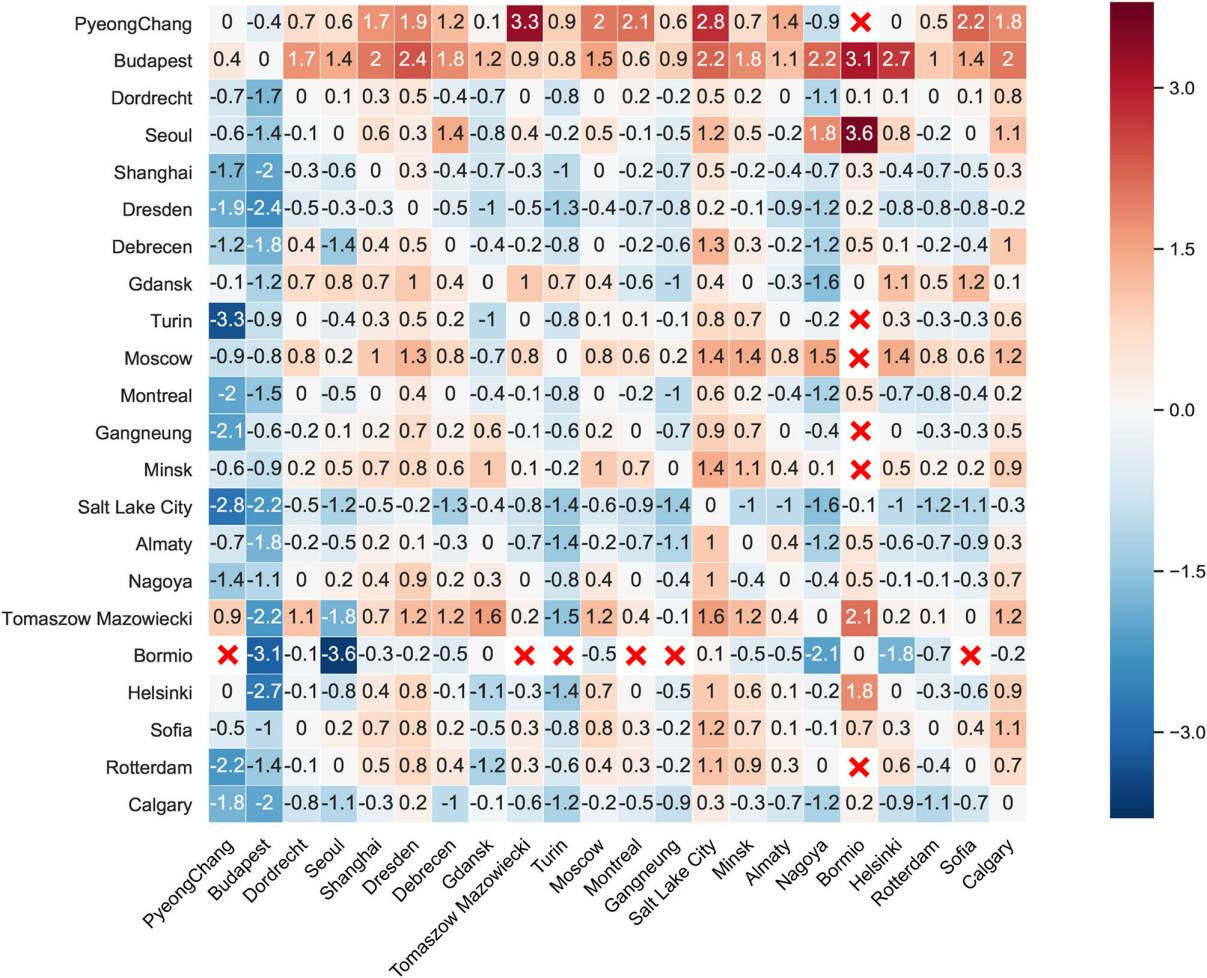
Performance-time differences between cities. Color bar indicates the range of time differences, while red cross marks represent abnormal performance times, which are removed. The color bar on the right side of the confusion matrix suggested the scale of the fastest finishing time differences (City on vertical axis minus City on horizontal axis), blue (red) represented that City on the vertical axis was faster (slower) than City on the horizontal axis. The unit for the measurements of performance time is second.

As shown in Figure 1, twenty-two cities were incorporated for visualization and listed in the same order in both horizontal and vertical axes, so that the values on the diagonal from left-top to right-bottom equaled zero. The performance–time difference matrix was anti-symmetric, meaning that every entry in the transposed matrix was negative compared with the original one. Note that zero was also existing in some places other than diagonal, i. e., the entry on the intersection of Gdansk and Bormio or Debrecen and Montreal, representing the infinitesimal performance–time difference (<0.1 s).

### Multivariate Regression Model for Performance Times and Geographical Factors

We further incorporated the four geographical factors, longitude, latitude, altitude, and barometric pressure into a multivariate linear regression model to explain how performance time was affected by ice rink locations. Meanwhile, a collinearity test was conducted primarily to exclude redundant variables, especially for altitude and barometric pressure. The parameters in the initial regression model (Table 1) showed that the goodness-of-fit R^2^ was 0.574, representing the model fitted data well, while F < 0.05 meant the model was predictive. However, significant levels of barometric pressure and altitude were much higher than 0.05, and VIF values, which measured the collinearity, were also abnormally high (without collinearity when VIF value was below 10), implying that there was a high magnitude of collinearity level between barometric pressure. According to the beta values in the test model variables (Table 1), barometric pressure (1.256) was more important than altitude (0.680) to account for the performance time. As a result, latitude, longitude, and barometric pressure were finally involved in the modified multivariate regression model in Table 1, which could be described as follow,


(1) $$\begin{array}{l} Pd=0.014\times LAd+0.002\times LOd+0.059\times BPd+\;\\ \;6.692\times 10^{-17} \end{array}$$

where *pd*, *LAd*, *LOd*, and *BPd* represented the difference in performance times, latitudes, longitudes, and barometric pressures, respectively. Longitude is categorized as an input variable to probably indicate other geographical factors on different hemispheres that are hard to quantify, such as climate, ocean currents, etc.

**Table 1:** Parameters of test regression model and final regression model.

**Initial regression model (All variables taken)**				
**R**	**R** ^**2**^		**Adjusted R** ^**2**^	**F**
0.758	0.574		0.561	0.000
**Variables**				
	**B**	**Beta**	**Significant level**	**VIF**
Constant	7.075 × 10^−17^	-	1.000	-
Latitude	0.016	0.120	0.088	1.497
Longitude	0.002	0.221	0.02	2.716
Barometric pressure	0.127	1.256	0.363	582.746
Altitude	0.001	0.680	0.629	604.908
**Final regression model (Longitude, latitude, and barometric pressure)**				
**R**	**R** ^**2**^		**Adjusted R** ^**2**^	**F**
0.757	0.573		0.564	0.000
**Variables**				
	**B**	**Beta**	**Significant level**	**VIF**
Constant	6.962 × 10^−17^	-	1.000	-
Latitude	0.014	0.108	0.100	1.309
Longitude	0.002	0.204	0.02	2.344
Barometric pressure	0.059	0.590	0.000	2.263

*The values of B and beta are denoted as the regression and standardized regression coefficients, respectively*.

## Discussion

This study rigorously evaluated the player performance in short-track speed skating from geographical race factors due to different venue locations, which was a novel perspective that few works of literature reported. Consequently, we confirmed that ice rink locations could affect performance time in 500-m events through one-way ANOVA and presented the reference time for different hosting cities (Figure 1). The 500-m events were considered because of the relatively short distance (four and a half laps) compared to 1,000 or 1,500-m competitions. Hence the performance time was less influenced by the pacing strategy concerned with energy distribution.

The performance-time difference matrix showed that reference times ranged from ±3.6 s for the listed twenty-two cities (Figure 1), indicating that venue locations could boost or impair performance time to a large extent. Note that not all reference times were deduced from geographical race factors, and other geographical factors such as home advantages or time zones also affected the value. Another particular remark is that the physical properties of ice (hardness and structure) have potential effects on athletic performance because they influence the contact area between blades and ice surfaces (Poirier et al., 2011a). Ice hardness affects friction, with higher values for a smooth surface than for rough ones (Rohm et al., 2015), which results in an increase in the contact area. Meanwhile, ice structure determines the melting point (Rohm et al., 2015) that causes variations in the coefficient of friction (Poirier et al., 2011b). Besides, values in performance–time difference matrix were not transitive since skaters who competed in both venues were different.

The practical applications of our findings indicate that elite skaters could comprehensively consider their personal performance and reference time according to the city where the competition was held, which aids to set up a goal to win the championship, and is of particular help during training. The term “reference time” is a novel concept, which serves as a solution to guide allocations of stamina and pacing strategies since one major challenge for skaters is to adopt flexible pacing behaviors in competitions. Notably, reference time at Salt Lake City is uniformly lower than in any other city, which is consistent with the previous conclusion that higher altitudes led to greater performance time (van Ingen Schenau, 1982; Koning, 2005). Also, Budapest presents uniformly larger reference time inferring that higher barometric pressure and lower altitude accounted for this phenomenon.

The use of machine learning models to visualize and analyze data gains favor in modern sports science (Guo et al., 2020) due to its outstanding abilities for generalization and efficiency in the investigation of complex latent interactions between variables. We established a multivariate linear regression model to investigate how altitude, barometric pressure, longitude, and latitude affected performance time. Barometric pressures were highly correlated with altitude, however, the margins between them were small. Therefore, we only used data with values >7 kPa to amplify the effects. The obtained regression model indicated that longitude, latitude, and barometric pressure were the key factors to performance-time difference since altitude was collinear with barometric pressure. The coefficients of latitude were large, meaning it was the most sensitive variable in the model. An interesting note inferred from the multivariate regression model was that skaters could ignore the eastern or western hemispheres but should check the air pressure and latitude of the venues before they participate in the upcoming competitions.

In conclusion, this study used quantitative techniques to analyze how geographical race factors of ice rinks affected performance time in short-track speed skating. Nonetheless, the limitations of this study still existed, such as the sample size being insufficiently large and only 500-m events were considered. Additionally, the self-built dataset was non-discriminated between open-air and closed venues since the potential impact of wind direction in open-air arenas is unignorable. All of these limitations deserve thorough modifications in future works.

## Data Availability Statement

The raw data supporting the conclusions of this article will be made available by the authors, without undue reservation.

## Author Contributions

LS and KT designed the study. KT supervised the study. TG and FL performed the experiments. LS, TG, and KT wrote the manuscript. All authors contributed to the article and approved the submitted version.

## Funding

This study was supported by grants from the National Key Research and Development Program of the Ministry of Science and Technology, China (2020YFF0304700).

## Conflict of Interest

The authors declare that the research was conducted in the absence of any commercial or financial relationships that could be construed as a potential conflict of interest.

## Publisher's Note

All claims expressed in this article are solely those of the authors and do not necessarily represent those of their affiliated organizations, or those of the publisher, the editors and the reviewers. Any product that may be evaluated in this article, or claim that may be made by its manufacturer, is not guaranteed or endorsed by the publisher.
